# mRNA Vaccines as Novel Immunotherapeutic Approaches in Gastrointestinal Cancers

**DOI:** 10.3390/cells15181673

**Published:** 2026-09-16

**Authors:** Michele Ghidini, Federica Novazzi, Nerina Denaro, Angelica Petrillo, Margherita Ratti, Massimiliano Salati, Roberta Malvaso, Lorenza Di Marco, Alessandro Parisi, Jens Claus Hahne

**Affiliations:** 1 Medical Oncology Division, ASST Sette Laghi, 21100 Varese, Italy; 2 Department of Medicine and Technological Innovation (DiMIT), University of Insubria, 21100 Varese, Italy; federica.novazzi@uninsubria.it; 3 Laboratory of Medical Microbiology and Virology, ASST Sette Laghi, 21100 Varese, Italy; 4 Department of Medical Oncology, Fondazione IRCCS Ca’ Granda Ospedale Maggiore Policlinico, 20122 Milan, Italy; nerina.denaro@policlinico.mi.it; 5 Medical Oncology Unit, Ospedale del Mare, 80147 Naples, Italy; angeli.petrillo@gmail.com; 6 Oncology and Hematology Department, Piacenza General Hospital, 29121 Piacenza, Italy; mratti.cremona@gmail.com; 7 Modena Cancer Centre, University Hospital of Modena, 41124 Modena, Italy; salati.massimiliano@aou.mo.it (M.S.); lor.dimarco@gmail.com (L.D.M.); 8 Department of Oncology, Università Politecnica delle Marche, 60121 Ancona, Italy; robertamalvaso1410@gmail.com (R.M.); alexparis@hotmail.it (A.P.); 9 NIHR Biomedical Research Centre Leicester, University of Leicester, Leicester LE3 9QP, UK

**Keywords:** mRNA vaccines, gastrointestinal cancers, neoantigens, immunotherapies, lipid nanoparticles, liposomes, immune checkpoint inhibitors, tumour microenvironments

## Abstract

Messenger RNA vaccines have moved rapidly from the infectious-disease area into oncology. They now represent an attractive therapeutic option for gastrointestinal (GI) cancers. Personalised constructs, designed to elicit tumour-specific T-cell immunity, have shown acceptable safety and clear immunogenicity in early-phase trials of colorectal and pancreatic cancers. These early signals are encouraging, but several obstacles still exist for routine clinical use: tumour heterogeneity, immune evasion, the difficulty of selecting the right target antigens, and the stability of the delivery platform itself. The possibility of a combination with immune checkpoint inhibitors is currently being explored to deepen and prolong responses, although the durability of benefit remains to be established. In this review, we outline the current landscape of mRNA vaccine development for GI malignancies, with particular attention to delivery strategies and multimodal immunotherapy. Finally, we discuss the directions most likely to help move these agents from the laboratory into everyday clinical practice.

## 1. Introduction

Gastrointestinal (GI) tumours comprise cancers of the digestive system, including colorectal, gastric, and pancreatic cancer. These tumours are among the most frequently diagnosed malignancies worldwide and account for a substantial share of cancer-related morbidity and mortality [1]. Conventional treatment, based on surgery, chemotherapy, and radiotherapy, offers only limited benefit in advanced disease. Although targeted agents and immune checkpoint inhibitors (ICIs) have improved outcomes, this benefit has so far been confined to selected subgroups of patients.

The main obstacle to current immunotherapy is primary or acquired resistance, which is especially pronounced in so-called “cold” tumours. Cold tumours are characterised by a low tumour mutational burden (TMB), sparse T-cell infiltration, and a strongly immunosuppressive tumour microenvironment (TME) [2,3]. These features are most evident in pancreatic ductal adenocarcinoma (PDAC) and in microsatellite-stable colorectal cancer (MSS-CRC), two settings in which the need for new immune-priming strategies is particularly acute [4,5].

Therapeutic tumour vaccines have re-emerged in this context because of their capacity to engage the immune system and, at least in principle, to generate durable antitumour responses [6]. The recent convergence of nanotechnology and synthetic nucleic-acid chemistry has turned RNA from a fragile, transient messenger into a designable molecular tool for cancer therapy [7]. A range of RNA-based approaches has been investigated, including antisense oligonucleotides, small interfering RNA, microRNA modulators, messenger RNA (mRNA) therapeutics, aptamers, short hairpin RNA, and CRISPR/Cas single-guide RNAs [8].

Among these, mRNA vaccines have attracted the most interest, mainly because they can be designed, manufactured, and adapted to encode virtually any antigen within a short time frame [8,9]. In practical terms, an mRNA vaccine consists of a synthetic mRNA encoding either tumour-specific antigens (TSAs) or tumour-associated antigens (TAAs). TAAs are proteins expressed by both malignant and normal tissue that the immune system can nevertheless perceive as foreign, whereas neoantigens are unique products of tumour-specific genetic alterations [10,11]. After delivery into host cells, usually inside liposomes (hollow aquous core with lipid bilayer) or lipid nanoparticles (LNPs), the mRNA is translated into antigenic protein, which is then processed and displayed on MHC class I and II molecules for recognition by T-cells. This drives both CD8^+^ cytotoxic and CD4^+^ helper T-cell responses directed against tumour cells expressing the relevant antigen [12,13].

Several conditions favour an effective response: a high TMB, a pool of functional and non-exhausted tumour-infiltrating lymphocytes (TILs), adequate antigen presentation, and high-level expression of the target TAAs. GI tumours frequently fail to meet these conditions, and their cold, immunosuppressive microenvironment limits the effect of both chemotherapy and immunotherapy [2,3]. Although it is important to note that mRNA vaccines were already being evaluated in clinical trials in patients before the COVID-19 pandemic—with the first human clinical trial results published in 2008 [14]. The pandemic undoubtedly accelerated the maturation of mRNA technology and its manufacturing infrastructure, which has created additional momentum to address these barriers [15].

One practical limitation deserves emphasis at the outset. Personalised mRNA cancer vaccines derived from a patient’s own tumour require a long manufacturing chain: tumour biopsy, sequencing and neoantigen identification, selection and design of the mRNA constructs, DNA template synthesis, in vitro transcription, mRNA purification and quality control, liposome or LNP encapsulation, and finally sterility testing and release [7]. Depending on the manufacturing capacity, regulatory requirements, and quality control, the whole process can take from several weeks to a few months. This turnaround is a real problem in rapidly progressing tumours such as PDAC, where the disease may advance faster than the vaccine can be produced. Table 1 summarises the main advantages, limitations, and potential applications of mRNA vaccines versus other therapeutic cancer-vaccine platforms, which is relevant to gastrointestinal tumours.

**Figure 1:**
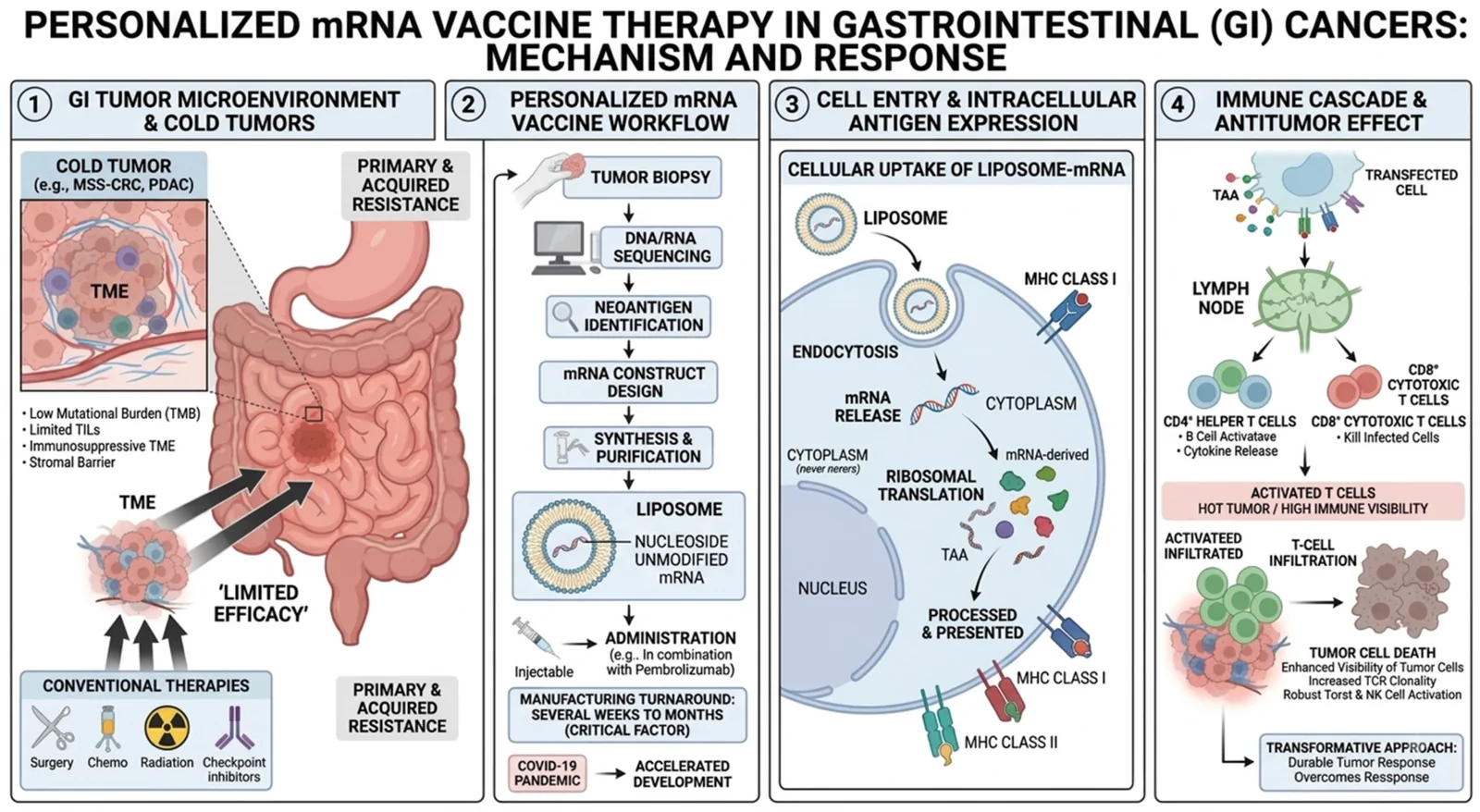
Overview of personalised mRNA vaccine therapy in gastrointestinal (GI) cancer. The schematic summarises, from left to right: (1) the immunosuppressive “cold” tumour microenvironment typical of GI malignancies such as MSS colorectal cancer and pancreatic ductal adenocarcinoma, as well as the limited efficacy of conventional therapy; (2) the personalised mRNA vaccine workflow, from tumour biopsy and sequencing through neoantigen identification, construct design, synthesis, and encapsulation of nucleoside-unmodified mRNA (liposome is shown here as an example) to administration; (3) cellular uptake of the encapsulated mRNA, cytoplasmic translation, and antigen processing and presentation on MHC class I and II molecules; (4) the resulting immune cascade, in which primed CD8^+^ cytotoxic and CD4^+^ helper T-cells drive tumour-cell killing and convert a “cold” into a “hot” immunologically active lesion. Legend: CRC: colorectal cancer; MSS: microsatellite-stable; PDAC: pancreatic cancer; TAA: tumour-associated antigen; TME: tumour microenvironment.

Figure 1 provides an integrated overview of the whole process, from the cold GI tumour microenvironment through the personalised vaccine workflow to the resulting antitumour immune response.

The choice of antigen, whether a shared TAA or a private neoantigen, is therefore critical in a GI setting. Furthermore, the delivery vehicle is extremely important because naked mRNA is quickly degraded by ubiquitous nucleases. Therefore, it must be protected and delivered efficiently, and this is achieved by encapsulation either within liposomes or LNPs. LNPs are composed of an ionizable lipid, a helper lipid, cholesterol, and a PEG-lipid, which shield the mRNA and promote its extravasation and cellular uptake [7,23]. Nanovaccine platforms are usually grouped into polymer, inorganic, liposomal, and cell-based nanoparticle formulations [7]. Reflecting the momentum in this area, several studies presented at the 2026 AACR Annual Meeting reported robust immune responses to mRNA nanovaccines encoding membrane cytokines together with shared or mutated antigens [8,24,25]. These constructs deliver the genetic template for intracellular antigen expression, so that processing and MHC presentation occur endogenously and no exogenous protein handling is required.

In this review, we focus on the immunomodulatory mechanisms of mRNA vaccines and the immunotherapeutic strategies built around them in GI cancers. We then consider the principal obstacles, together with the ongoing efforts to optimise vaccine design, delivery, and stability.

## 2. Mechanisms of Action: Biology and Functions of mRNAs

Messenger RNA is a single-stranded nucleic acid that acts as the genetic intermediary carrying protein-coding information from the nuclear DNA to the cytoplasmic ribosomes, where it is translated into functional protein [26]. During transcription, RNA polymerase II binds specific DNA sequences and synthesises a pre-mRNA complementary to the template strand. This transcript is then processed through 5′ capping, splicing, and 3′ polyadenylation to form the mature mRNA that leaves the nucleus through the nuclear pores. The mature molecule carries its coding information as codons (triplets of the four nucleotides (A, U, C, G)). At the ribosome, the sequence is read codon by codon while transfer RNAs bearing the matching anticodons deliver the corresponding amino acids to build the growing polypeptide chain. Beyond serving as a template for protein synthesis, mRNA also helps to regulate how much protein a gene produces, through transcription rate, transcript stability, and translational efficiency. This effectively links the genetic code to the actual assembly of cellular proteins [26].

Using mRNA as a vaccine is a comparatively recent idea, even though it has been known since the early 1990s that injected mRNA can drive antigen expression and induce antigen-specific cytotoxic T-cells [27,28]. Notably, these early studies used unmodified mRNA containing standard uridine. In 2000, this concept was confirmed by showing that mRNA immunisation could generate cellular and humoral responses at least as effective as DNA [29]. Importantly, mRNA vaccines allow the host cell itself to express the antigen, including transmembrane proteins and viral glycoproteins that carry their natural glycosylation pattern [30].

Recent work has clarified several cellular steps that govern how synthetic mRNA behaves once it is administered. Heparan sulphate at the cell surface assists in mRNA uptake, while the vacuolar ATPase acidifies the endosome to release mRNA into the cytoplasm. Sometimes N1-methylpseudouridine was incorporated to prevent TRIM25-mediated degradation of the transcripts and to improve its stability and therapeutic activity [31,32]. However, nucleoside modification is not a requirement for an effective mRNA vaccine. Several cancer vaccines rely instead on unmodified, canonical uridine mRNA, including BioNTech’s individualised neoantigen vaccine BNT122 (autogene cevumeran) [18,33], and self-amplifying mRNA (saRNA) platforms—such as the approved COVID-19 vaccine zapomeran (KOSTAIVE) and the GRANITE-CRC-1L trial [34]. The use of unmodified nucleotides is required because the encoded viral replicase does not efficiently recognise modified bases. In general, unmodified mRNA may in fact be preferred for cancer vaccines because it preserves a degree of innate type I interferon signalling that contributes to antitumour immunity [35]. Furthermore, N1-methylpseudouridine has been shown to cause ribosomal slippage and +1 frameshifting, generating off-target translation products that are undesirable in a personalised cancer vaccine [36]. These findings help to explain how mRNA therapeutics bypass the cell’s own defence mechanisms. They pave the way towards more efficient RNA-based treatments for both cancer and inherited disease [9,37]. The same approach has been exploited to correct genetic disorders by supplying a functional transcript to cells that lack it, as well as to build vaccines in which the encoded antigen triggers a protective immune response [9].

Compared with DNA vaccines, a mRNA-based vaccine has the practical advantage of only needing to reach the cytoplasm to be translated, so delivery is simpler. Because there is no reverse transcriptase to copy the transcript back into DNA, there is also no risk of integration into the host genome [38]. The overall risk profile is favourable, since production requires no cultivation of pathogens or handling of infectious material at any stage. All that is needed is the genetic sequence, which is now readily available from online databases [38]. In addition, only a limited number of antigens are expressed for a short time. RNA was long regarded as too unstable to serve as a genetic vector but working in an RNase-free environment and formulating the transcript appropriately now make it possible to produce stable, even thermostable, mRNA vaccines [39].

A practical approach to therapeutic mRNA vaccine development follows a staged workflow that links antigen discovery, vaccine manufacture, three-dimensional testing, and in vivo validation. Tumour and normal tissues are first sequenced to identify expressed patient-specific mutations, which are ranked according to predicted antigen presentation and immunogenicity. Selected neoantigens are then encoded in one or more optimised mRNA transcripts and formulated into LNPs or lipoplexes (complex structure formed by the electrostatic association of positively charged cationic or ionizable lipids and negatively charged mRNA) to promote uptake by antigen-presenting cells [16,17]. In a phase I pancreatic cancer study, for example, up to 20 patient-specific neoepitopes were encoded in two mRNA strands and delivered as a lipoplex vaccine. A vaccine-induced T-cell response were detected in eight of sixteen patients and were associated with delayed recurrence [18]. Three-dimensional models can provide an intermediate step between molecular design and animal testing by assessing delivery, tissue penetration, antigen expression, immune cell recruitment, and tumour-cell killing under more realistic spatial conditions than conventional two-dimensional cultures. A 3D melanoma co-culture demonstrated that RNA-lipoplex penetration and translation were strongly influenced by tissue architecture and cell type, effects that were not evident in 2D cultures [40]. Similarly, patient-derived CRC organoids co-cultured with dendritic cells in a collagen matrix allow the analysis of dendritic cell migration, tumour fragment uptake, activation marker expression, and T-cell priming capacity [41]. Although this CRC model has not yet been used directly to test mRNA vaccines, it provides a suitable platform for evaluating whether an mRNA formulation reaches dendritic cells and restores antigen presentation within an immunosuppressive tumour environment. Promising candidates can then be advanced to syngeneic, patient-derived, or humanised mouse models to assess biodistribution, systemic immune activation, tumour control, immunological memory, and toxicity before clinical translation [22]. Thus, the purpose of combining mRNA vaccines with 3D culture and preclinical models is not simply to reproduce clinical treatment in miniature, but to progressively test whether the vaccine is correctly delivered, biologically active, immunologically functional, and effective within increasingly complex tumour environments.

A mRNA vaccine consists of a synthetic transcript with five key elements (Table 2) [42,43]. Together, these components determine how efficiently and how durably the antigen is expressed.

Delivery relies on liposomes or LNPs, which protect the transcript within the body and promote its entry into cells [44,45].

Once the mRNA reaches the cytoplasm, ribosomes translate it into antigen. Afterwards, the transcript is degraded. Neither the mRNA nor the transcript enters the nucleus or alters the host DNA. This combination—cytoplasmic translation, absence of genomic integration, and natural degradation within a few days—is the basis of the platform’s favourable safety profile, while the expressed antigen stimulates both humoral and cellular immunity.

The mRNA and the protective delivery vehicle both contribute to the innate response, but in different ways [42,46]. Liposomes as well as LNPs can induce inflammatory cytokines such as IL-6 in fibroblasts at the injection site, whereas the mRNA itself drives production of type I interferons (e.g., IFN-β) by activating dendritic cells and recruiting monocytes [42,46]. The adaptive response has two arms. The first is a humoral one, in which mRNA vaccines can generate neutralising antibody titers exceeding those in convalescent serum [47]. The second is a cellular one dominated by Th1-polarised CD4^+^ and cytotoxic CD8^+^ T-cells [43,48]. The result is a coordinated response—an IFN-driven innate component together with a durable antibody and T-cell immunity—that underlies the long-lasting protection seen with this platform [43,44]. In an orthotopic CRC model, intramuscular LNP administration activated dendritic cells in draining lymph nodes, while all-trans-retinoic acid induced CCR9 and α4β7 expression on antigen-specific T-cells and promoted their migration to intestinal tumours [49]. Thus, therapeutic activity may depend primarily on dendritic-cell activation, T-cell priming, and mucosal trafficking. LNPs and modified mRNA should therefore be presented as important but not universal components of anticancer mRNA vaccine design.

**Figure 2:**
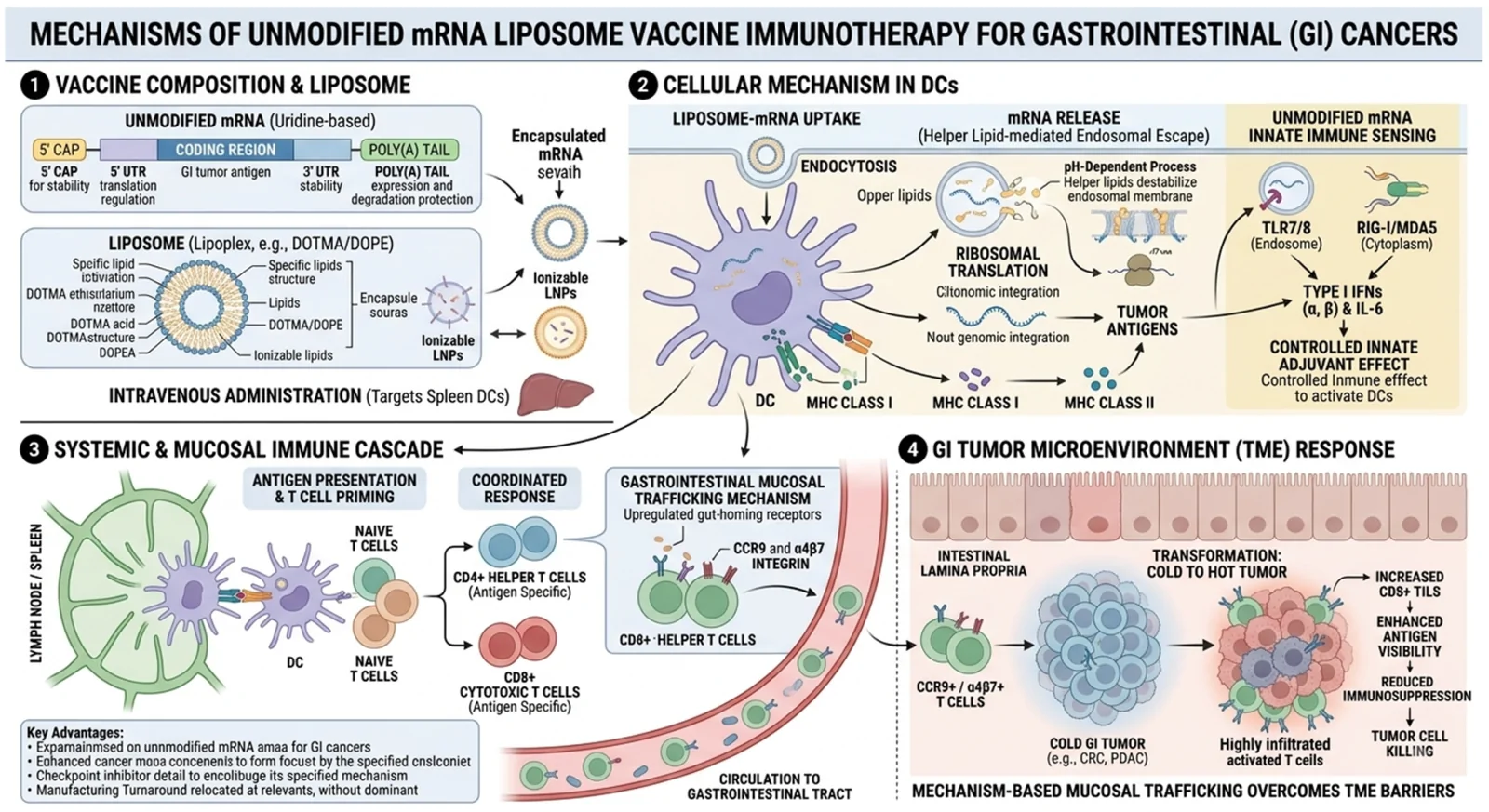
Mechanisms of unmodified mRNA liposome vaccine immunotherapy for gastrointestinal (GI) cancers. 1. Vaccine composition and liposome: Unmodified mRNA encoding GI tumour antigens is encapsulated in ionizable liposomes (DOTMA/DOPE) and administered intravenously to target spleen dendritic cells (DCs). 2. Cellular mechanism in DCs: Liposomes enter DCs via endocytosis, releasing mRNA through pH-dependent endosomal escape. Ribosomes translate mRNA into tumour antigens presented on MHC class I and II. Simultaneously, mRNA triggers TLR7/8 and RIG-I/MDA5 pathways, inducing type I IFNs and IL-6 to activate DCs. 3. Systemic and mucosal immune cascade: Activated DCs present antigens to naive T-cells in the spleen/lymph nodes, priming CD4^+^ helper and CD8^+^ cytotoxic T-cells. These T-cells upregulate gut-homing receptors (CCR9 and α4β7 integrin) and enter systemic circulation. 4. GI tumour microenvironment (TME) response: Homing T-cells infiltrate the intestinal lamina propria, transforming “cold” tumours (e.g., CRC, PDAC) into highly infiltrated “hot” tumours. This increases CD8^+^ TILs, enhances antigen visibility, reduces immunosuppression, and triggers tumour-cell killing. Legend: CRC: colorectal cancer; DC: dendritic cell; IL6: interleukin 6; PDAC: pancreatic ductal adenocarcinoma.

In summary, mRNA vaccines offering several advantages, e.g., high potency, an acceptable safety and efficacy profile, rapid development against new targets, scalable and relatively inexpensive manufacturing, and the option of multi-epitope designs [50,51]. Most current manufacturing pipelines rely on in vitro transcription (IVT) to generate the mRNA construct. However, the chemical synthesis of mRNA is emerging as an alternative production route that may shorten and simplify manufacturing, particularly for individualised cancer vaccines [52]. Nevertheless, some limitations exist like the need for cold-chain logistics because of transcript instability, the difficulty of directing delivery to specific tissues or cells, the cost of mRNA encapsulation, and the requirement for careful monitoring of rare adverse events [50,51]. The main steps of this process, from vaccine structure and cell entry to the coordinated innate and adaptive responses, are summarised in Figure 2.

## 3. Immunomodulatory and Antitumour Effects of mRNA Vaccines

Cancer vaccines have emerged as a promising way to induce tumour-specific immunity by delivering TAAs or neoantigens [6]. As mentioned before, mRNA-based vaccines have attracted particular attention because they are versatile, quick to manufacture, and generally well tolerated [10,44]. Unlike DNA-based approaches, mRNA does not need to enter the nucleus and carries no risk of genomic integration, yet it can direct expression of essentially any antigen, including patient-specific neoantigens [11]. Improvements in delivery, together with nucleoside modification in some platforms or the use of unmodified, canonical uridine mRNA in others (see Section 2), have further enhanced the stability, translation, and immunogenicity of the transcript [23,44]. In GI cancers specifically, mRNA vaccines offer a way to counter immune resistance by inducing de novo antitumour responses, improving antigen presentation, and reshaping the TME [53,54].

After administration as liposome or LNP formulation, the mRNA vaccine construct is preferentially taken up by professional antigen-presenting cells, above all dendritic cells in lymphoid tissue [12,13]. The transcript is translated into TAAs or neoantigens, which are then processed through both MHC class I and class II pathways, allowing cross-presentation and priming of CD8^+^ cytotoxic and CD4^+^ helper T-cells [12,13,55,56]. Design features such as codon optimisation, stabilisation of the untranslated regions, and, where used, nucleoside modification improve translation while limiting excessive innate sensing of the transcript [57,58,59].

At the innate level, mRNA vaccines have an intrinsic adjuvant effect [60,61]. They engage pattern-recognition receptors, including Toll-like receptors and cytosolic RNA sensors, which activate the interferon regulatory factor and NF-κB pathways and lead to type I interferons and inflammatory cytokines production [60,61,62]. This promotes dendritic-cell maturation and T-cell priming, but it must be kept within limits, since excessive sensing can inhibit translation or drive tolerogenic signalling [58]. Preclinical work has also shown that mRNA vaccination can trigger epitope spreading, broadening the response beyond the antigen originally encoded [12,63].

GI malignancies typically present an immunosuppressive TME, enriched in regulatory T-cells, myeloid-derived suppressor cells, and M2-like tumour-associated macrophages, and shaped by inhibitory cytokines such as TGF-β and IL-10 [3,64]. mRNA vaccines can partly reverse this state by increasing CD8^+^ T-cell infiltration, raising interferon-γ production, and repolarising macrophages towards an M1 phenotype [2,65]. Next-generation constructs that also encode cytokines or co-stimulatory ligands can push T-cell expansion further and help to counter exhaustion, an effect that is most apparent when they are combined with ICIs [66,67].

Early-phase trials have confirmed that mRNA cancer vaccines are immunogenic and safe in GI malignancies. One example is BioNTech’s personalised neoantigen vaccine BNT122 (RO7198457). It induces robust neoantigen-specific CD4^+^ and CD8^+^ T-cell responses in patients with advanced solid tumours [12,68,69]. The combination of BNT122 with PD-1 blockade has produced synergistic immune activation even in microsatellite-stable colorectal cancer [4,70]. A further pivotal study showed that a personalised mRNA vaccine (autogene cevumeran) given after resection of pancreatic cancer induced durable neoantigen-specific T-cell responses and delayed recurrence. Responders displayed expansion of distinct T-cell receptor clonotypes and long-lived memory responses, which points to genuine and sustained immunological control [18,33].

In parallel, off-the-shelf vaccines directed at shared antigens such as carcinoembryonic antigen and mutant KRAS are currently being tested [71,72]. Numerous ongoing trials are evaluating mRNA vaccines in GI cancers together with ICIs, chemotherapy, or radiotherapy [11,73]. Emerging evidence suggests that TMB, neoantigen load, and baseline immune infiltration may all influence the likelihood of a response [74,75]. At the bench, single-cell sequencing, spatial transcriptomics, and T-cell receptor profiling have illuminated the immune dynamics that follow vaccination. An expansion of neoantigen-specific clones, higher intratumoural CD8^+^ T-cell density, and activation of interferon signalling was consistently shown [76,77,78,79]. Taken together, these findings establish mRNA vaccines as a credible strategy in GI oncology, able to induce antigen-specific immunity and remodel the TME.

### 3.1. Delivery and Antigen Presentation

After administration, mRNA vaccines are most often delivered as LNPs or liposomal formulations, although lipoplexes and related carriers have also been used clinically. Biodistribution depends on particle composition, size, surface properties, and route of administration. The uptake is therefore not restricted to dendritic cells but may also involve macrophages, stromal cells, and other antigen-presenting cells at the injection site and in draining lymphoid tissue [23,44]. Following endosomal escape, the transcript is translated in the cytoplasm without entering the nucleus or integrating into the host genome, which underlies the favourable safety profile of this platform.

The translated tumour antigen can enter several antigen-presentation pathways. Cytosolic processing generates peptides for MHC class I presentation and activation of CD8^+^ cytotoxic T-cells, while antigen transfer, autophagy-associated routing, or the incorporation of lysosomal targeting sequences can promote MHC class II presentation and CD4^+^ T-cell activation. Furthermore, dendritic cells can also cross-present extracellular or cell-associated vaccine antigen on MHC class I [19,55,56]. An effective GI-cancer construct should therefore ideally stimulate both CD8^+^ effector cells and CD4^+^ helper cells rather than favour a single arm of the response.

The molecular structure of the transcript affects this balance. The 5′ cap, untranslated regions, poly(A) tail, codon usage, and nucleoside composition all influence stability, translation efficiency, degradation kinetics, and innate immune sensing [57,58,59]. Nucleoside modification can increase protein output by reducing recognition of the transcript, whereas unmodified uridine can provide stronger intrinsic adjuvanticity at the cost of lower translation. In a comparative melanoma model, unmodified-uridine mRNA induced stronger type I interferon production, dendritic-cell maturation, and polyfunctional antitumour CD8^+^ T-cell responses than a highly modified formulation. However, this result was model- and formulation-dependent and should not be interpreted as universal superiority of one chemistry over the other [35].

### 3.2. Innate Sensing and Dendritic-Cell Licencing

mRNA vaccines function as both antigen-delivery systems and innate immune adjuvants. RNA is detected by endosomal Toll-like receptors, particularly TLR3, TLR7, and TLR8, as well as by cytosolic sensors such as RIG-I and MDA5 [60,61]. These pathways activate MyD88-, TRIF-, or MAVS-dependent signalling, converging on IRF3, IRF7, and NF-κB activation and leading to the production of type I interferons, IL-6, IL-12, TNF-α, and other inflammatory mediators [19,80]. This innate response promotes dendritic-cell maturation by increasing expression of antigen-presentation and co-stimulatory molecules, including MHC class I, MHC class II, CD40, CD80, and CD86, after which mature dendritic cells migrate to draining lymph nodes to prime naive T-cells [62].

The magnitude and location of RNA sensing are critical. Excessive activation can inhibit translation through interferon-induced antiviral pathways, accelerate RNA degradation, and promote unwanted tissue inflammation, whereas insufficient sensing may yield antigen expression without adequate dendritic-cell activation [58]. Work with RIG-I-activating immunostimulatory RNA has provided direct experimental evidence that controlled innate stimulation can enhance cross-presentation and CD8^+^ T-cell priming. In murine models, RIG-I activation increased dendritic-cell production of type I interferons and IL-12, enhanced antigen-specific T-cell expansion, and synergised with CTLA-4 blockade to produce durable antitumour immunity [80]. Alternative particle designs may additionally engage stromal cells and induce systemic chemokine production, illustrating that the relevant immunological target of a mRNA vaccine need not always be a dendritic cell [81].

### 3.3. Activation of Adaptive Antitumour Immunity

The adaptive response begins when vaccine-loaded dendritic cells present tumour-derived peptides to naive T-cells. MHC class I presentation favours expansion of CD8^+^ cytotoxic T-lymphocytes, which recognise and kill tumour cells through perforin and granzyme release, Fas–Fas ligand signalling, and production of interferon-γ and TNF-α. In contrast, MHC class II presentation activates CD4^+^ helper T-cells, particularly type-1 helper responses that support dendritic-cell licencing, IL-12 production, CD8^+^ T-cell expansion, and memory formation [63].

The importance of engaging both antigen-presentation pathways in GI malignancies was directly demonstrated in a CRC model, in which co-administration of MHC class I- and MHC class II-restricted neoantigen mRNAs generated stronger antigen-specific responses than either component alone. The combined vaccine increased CD8^+^ T-cell cytokine production, enhanced effector-cell development, reduced regulatory T-cell abundance, and improved tumour control [82]. All these effects were further strengthened by immune checkpoint blockade [82]. In clinical studies, mRNA vaccination has similarly induced de novo immunity against tumour mutations not detectable before treatment. In patients with metastatic GI cancers, a personalised mRNA vaccine generated mutation-specific T-cell responses in three of four treated patients, including both CD4^+^ and CD8^+^ responses and recognition of a KRAS G12D driver mutation. The treatment was well tolerated, but no objective clinical responses were observed, underscoring that immunogenicity alone does not guarantee tumour regression [69].

### 3.4. Remodelling of the Tumour Microenvironment

Many gastrointestinal tumours, particularly PDAC, contain an immune-excluded or immune-suppressed microenvironment characterised by poor dendritic-cell recruitment, limited T-cell infiltration, dense desmoplastic stroma, suppressive myeloid cells, regulatory T-cells, and inhibitory cytokines such as TGF-β and IL-10 [3,64]. mRNA vaccines can partly reverse this state through two complementary mechanisms. First, vaccine-induced interferon signalling promotes expression of chemokines such as CXCL9, CXCL10, and CXCL11, which attract activated T-cells into the tumour. In a cold-tumour model, an mRNA vaccine increased intratumoural CD8^+^ T-cell density, activated dendritic cells and macrophages, raised interferon-response gene expression, and generated a T-cell-inflamed microenvironment. Because vaccination also increased PD-1 and PD-L1 expression, combining the vaccine with anti-PD-1 therapy produced substantially better tumour control and survival than either treatment alone [83].

Second, mRNA constructs can be designed to deliver immunomodulatory proteins in addition to tumour antigens. In aggressive PDAC models, intratumoural mRNAs encoding IFN-α and IL-12, combined with chemotherapy and PD-1 and CTLA-4 blockade, recruited cDC1 dendritic cells and CD8^+^ T-cells, increased intratumoural granzyme B and interferon-γ, expanded effector-memory T-cells, and suppressed distant peritoneal metastases [84]. These results support the concept that antigen delivery and TME remodelling may need to occur together in poorly immunogenic GI cancers, although this cytokine-mRNA strategy was tested as part of a multimodal regimen and should not be interpreted as evidence that a neoantigen vaccine alone produces the same effects. Next-generation constructs that also encode cytokines or co-stimulatory ligands can further push T-cell expansion and help counter exhaustion, an effect most apparent when combined with ICIs [66,67].

### 3.5. Checkpoint Synergy, Exhaustion, and Immune Memory

mRNA vaccination and immune checkpoint inhibition act at complementary stages of the cancer-immunity cycle. Vaccination increases the number and diversity of tumour-reactive T-cells, while checkpoint blockade removes inhibitory signals that limit their expansion and function [2]. This complementarity is particularly relevant in GI cancers because interferon signalling and T-cell infiltration can simultaneously induce adaptive resistance through PD-1 and PD-L1 upregulation. In a murine gastric cancer model, neoantigen mRNA vaccination combined with anti-PD-1 therapy increased tumour-infiltrating neoantigen-specific CD8^+^ T-cells and expanded both progenitor-exhausted and intermediate-exhausted T-cell populations. The progenitor-exhausted subset retained proliferative capacity and provided a renewable source of effector cells during checkpoint treatment. This may explain the improved control of subcutaneous tumours and peritoneal metastases observed in this model [85].

The strongest clinical evidence for durable, checkpoint-independent immune memory comes from personalised vaccination with autogene cevumeran in resected PDAC, where eight of sixteen treated patients developed high-magnitude neoantigen-specific responses. Vaccine-expanded T-cell clones reached approximately 10% of circulating T-cells, included polyfunctional effector CD8^+^ cells, persisted for extended periods, and re-expanded after booster vaccination. In general, patients with detectable vaccine-expanded T-cells had longer recurrence-free survival than nonresponders, although the nonrandomised phase I design does not establish that vaccination caused this difference in outcome [18,33]. Epitope spreading should be described cautiously in this context. Some preclinical studies have reported immunity against antigens not encoded by the vaccine, but this phenomenon is not universal. The early gastrointestinal clinical study discussed above did not demonstrate increased responses against predefined neoantigens. Epitope spreading should therefore be presented as a possible downstream effect rather than a consistent property of mRNA vaccination [69,83].

### 3.6. Evidence Across Gastrointestinal Cancers

Overall, the evidence supports a stepwise mechanism in which mRNA vaccines deliver tumour antigens to antigen-presenting cells, activate innate RNA-sensing pathways, mature dendritic cells, enhance MHC class I and class II presentation, prime and expand tumour-specific CD8^+^ and CD4^+^ T-cells, increase chemokine-mediated T-cell recruitment to the tumour, counter immune suppression, improve checkpoint sensitivity, and, in a subset of patients, establish durable effector and memory responses. Early-phase trials have confirmed that mRNA cancer vaccines are immunogenic and safe in GI malignancies. One example is the personalised neoantigen vaccine BNT122 (RO7198457), which induces robust neoantigen-specific CD4^+^ and CD8^+^ T-cell responses in patients with advanced solid tumours [12,68,69]. The combination of BNT122 with PD-1 blockade has produced synergistic immune activation even in microsatellite-stable colorectal cancer [4,70]. A further pivotal study showed that the personalised mRNA vaccine autogene cevumeran, given after resection of pancreatic cancer, induced durable neoantigen-specific T-cell responses and delayed recurrence, with responders displaying expansion of distinct T-cell receptor clonotypes and long-lived memory responses that point to genuine and sustained immunological control [18,33].

In parallel, off-the-shelf vaccines directed at shared antigens such as carcinoembryonic antigen and mutant KRAS are currently being tested [71,72], and numerous ongoing trials are evaluating mRNA vaccines in GI cancers together with ICIs, chemotherapy, or radiotherapy [11,73]. Emerging evidence suggests that tumour mutational burden, neoantigen load, and baseline immune infiltration may all influence the likelihood of response [74,75]. Single-cell sequencing, spatial transcriptomics, and T-cell receptor profiling have illuminated the immune dynamics that follow vaccination, including expansion of neoantigen-specific clones, higher intratumoural CD8^+^ T-cell density, and activation of interferon signalling [76,77,78,79]. The clinical evidence to date remains strongest for immunogenicity rather than objective tumour regression. Personalised vaccination has produced durable immune responses in pancreatic cancer and mutation-specific responses in metastatic gastrointestinal cancers. Nevertheless, the clearest evidence for tumour-microenvironment remodelling and checkpoint synergy remains largely preclinical [18,33,69,82,85]. Taken together, these findings establish mRNA vaccines as a credible strategy in GI oncology, able to induce antigen-specific immunity and remodel the TME, while underscoring that future trials should measure not only response rate but also antigen presentation, T-cell clonality, tumour infiltration, progenitor-exhausted T-cell populations, cytokine signalling, and longitudinal immune memory. The mechanistic components of mRNA vaccine activity in GI cancers are listed in Table 3.

## 4. Rationales for mRNA Vaccines in Gastrointestinal Oncology

mRNA vaccines in GI cancers can stimulate and expand tumour-specific T-cell responses in tumours that are otherwise poorly immunogenic [8,86,87]. In colorectal cancer, only the small subset of MSI (microsatellite instability)-high tumours derive substantial benefit from immunotherapy, whereas most MSS/pMMR (proficient mismatch repair) tumours remain largely refractory to ICIs [88]. PDAC presents a related but distinct problem. A dense desmoplastic stroma and a profoundly immunosuppressive TME restrict immune-cell infiltration and confer deep resistance to the checkpoint blockade [18]. By actively priming cytotoxic T-lymphocytes against tumour antigens, mRNA vaccines may improve immune recognition and help convert these cold tumours into more inflamed, immunologically active lesions [8].

Preclinical and early clinical studies have shown that mRNA vaccines can induce durable neoantigen-specific CD8^+^ T-cell responses in GI malignancies, which supports their therapeutic potential [18]. They appear most promising in combination with other strategies, whether it be ICIs, chemotherapy, or additional immunomodulatory agents [89,90]. Such combinations may overcome resistance by simultaneously boosting antigen-specific T-cell activation and relieving local immunosuppression. Therefore, mRNA vaccines are increasingly studied as part of multimodal regimens designed to improve the efficacy of immunotherapy in GI cancers [8,18,89].

Another attractive feature of the mRNA vaccine is its versatility. Several antigens can be encoded in a single construct, so different tumour subclones are targeted at once and the marked molecular heterogeneity of many GI tumours is addressed directly [8,90]. The design can also be revised quickly as new molecular data emerge or as the molecular make-up changes due to the evolving disease. This fits well with the logic of precision oncology. Furthermore, it should be feasible to deploy vaccines in the adjuvant or minimal-residual-disease setting. In this scenario, reinforcing immune surveillance could help prevent recurrence after surgery or systemic therapy [91].

However, several difficulties remain, because neoantigen selection is far from trivial. Not every predicted neoantigen elicits an effective response [8,91]. The immunosuppressive TME of GI tumours, with its regulatory T-cells, myeloid-derived suppressor cells, inhibitory cytokines, and stromal barriers, can blunt the activity of vaccine-induced T-cells. There are also practical and economic constraints tied to personalised manufacturing, from sequencing and bioinformatic analysis to production timelines, that currently limit broader use [91].

Overall, mRNA vaccines are a fast-moving area in the treatment of GI cancer. Their specificity, adaptability, and capacity to raise tumour-specific immunity offer a way to address the therapeutic limitations of immunologically cold tumours [8,86,88,92]. Further optimisation and clinical validation are needed, but mRNA vaccine has already introduced a new paradigm in precision immuno-oncology and may substantially widen the role of immunotherapy [91]. The clinical applications across individual GI cancers, including ongoing trials, are discussed in the following section.

## 5. Combining mRNA Vaccines with Other Therapies

The rationale for combining mRNA vaccines with ICIs is straightforward. The vaccine expands cancer antigen-specific T-cells and drives their differentiation into effector cells, but these cells frequently acquire inhibitory markers. Blocking the PD-1/PD-L1 axis then sustains and prolongs the vaccine effect, so that the two interventions act synergistically [18].

The clearest demonstration to date that adding an mRNA vaccine to the checkpoint blockade can improve outcomes in the adjuvant setting is the phase IIb KEYNOTE-942 study (NCT03897881) for melanoma [93,94], while this combination has not been evaluated in the GI cancer field up to now.

The combination of mRNA vaccines with chemotherapy has been explored less often. The immunological rationale is that cytotoxic chemotherapy releases additional tumour antigens as cells die. The released antigens promote T-cell recruitment to the TME and lowers the risk of immune escape through antigen loss. Reduced immune escape is most valuable in tumours with a low mutational burden and a strongly immunosuppressive environment [89,95].

This principle is currently investigated in colorectal cancer [34]. The ongoing phase II/III GRANITE-CRC-1L trial (NCT05141721) evaluates an individualised neoantigen vaccine in newly diagnosed microsatellite-stable metastatic CRC. It is based on a self-amplifying mRNA and an adenoviral vector, combined with nivolumab and ipilimumab and added to fluoropyrimidine/bevacizumab, against fluoropyrimidine/bevacizumab alone. In the phase I portion, the vaccine plus ICIs produced a molecular response (a fall of at least 50% in ctDNA) in four of nine previously treated patients. This correlated with better OS and these results justified moving the approach into a first-line maintenance setting [34]. The underlying hypothesis is that the poor benefit from ICIs in MSS/pMMR disease reflects a shortage of neoantigen-specific T-cells and immune infiltration. An individualised mRNA vaccine may correct this deficit by inducing strongly tumour-specific CD8^+^ T-cells [34].

Vaccines can also be directed at proteins that drive tumour growth and angiogenesis. Autogene cevumeran (BNT122), a personalised uridine mRNA–lipoplex, has been tested in resected PDAC together with chemo- and immunotherapy. In early clinical trials, the vaccine activated durable, neoantigen-specific T-cell responses and delayed cancer recurrence in responders [18].

The interaction of mRNA vaccines with radiotherapy is still in its infancy. Preclinical data, mostly with non-mRNA vaccines so far, suggest that irradiation can amplify vaccine-induced immunity by attracting T-cells to the treated site. In addition, irradiated cells are more susceptible to T-cell killing [96]. Proof-of-principle was provided with a poorly immunogenic murine model of triple-negative breast cancer. Radiotherapy upregulated genes encoding immunogenic MHC-I and MHC-II neoepitopes, and a vaccine directed at these epitopes induced CD8^+^ and CD4^+^ T-cell responses that improved the efficacy of radiotherapy [97].

**Figure 3:**
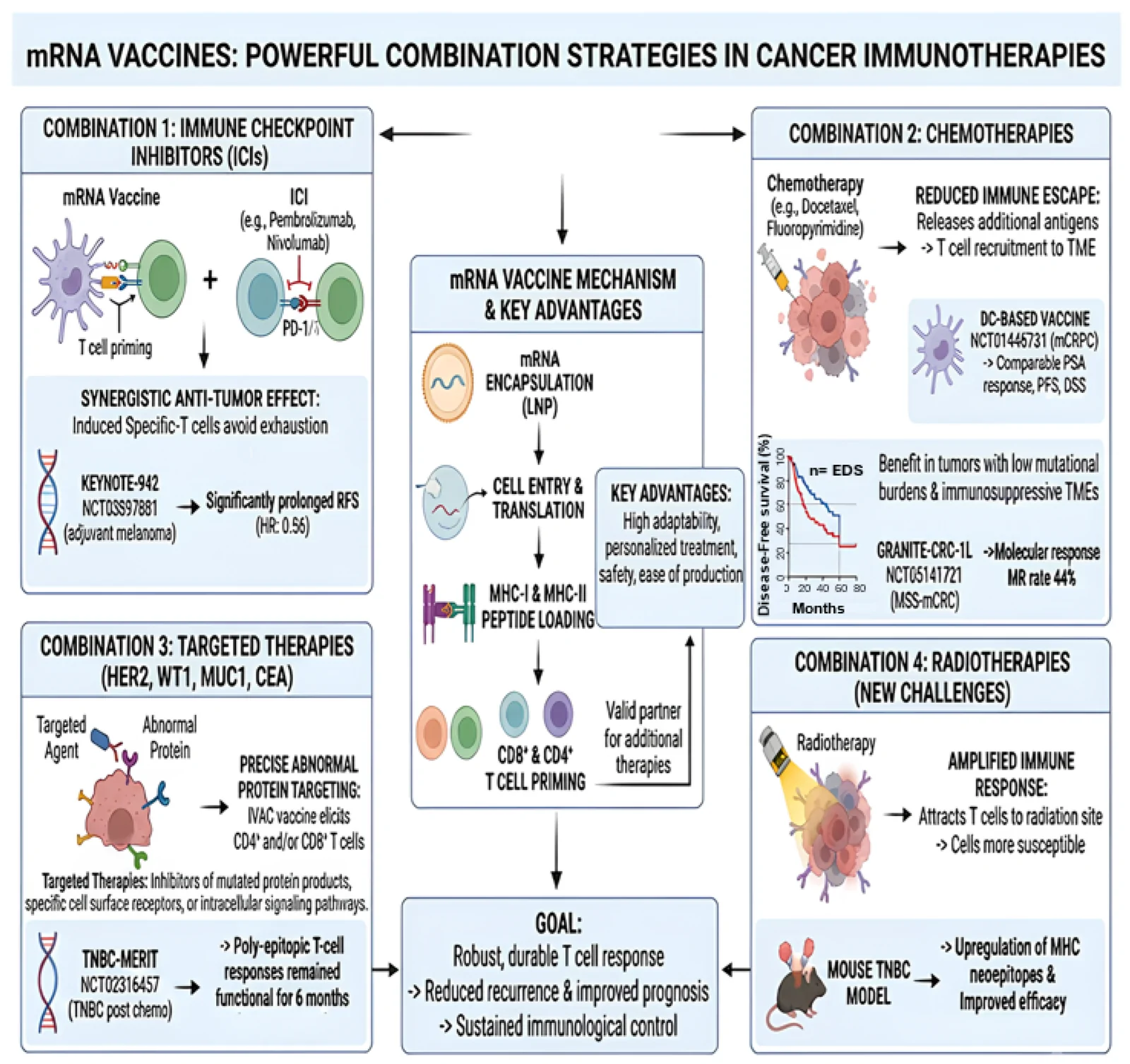
Combination strategies for mRNA cancer vaccines. Around the central mRNA vaccine mechanism and its key advantages [24], four combinatorial approaches are shown: (1) immune check-point inhibitors, which sustain vaccine-primed T-cells and prevent exhaustion (e.g., KEY-NOTE-942) [18,94]; (2) chemotherapies, which releases additional antigens and reduces immune escape (e.g., the DC-based vaccine trial and GRANITE-CRC-1L) [9,34,95]; Blue curve = patients receiving the experimental treatment (e.g., the mRNA vaccine strategy). Red curve = patients in the comparison/control group; (3) targeted therapies against tumour-associated antigens such as HER2, WT1, MUC1 and CEA (e.g., TNBC-MERIT) [89]; (4) radio-therapies, which amplifies the antitumour immune response. The shared goal is a robust, durable T-cell response, reduced recurrence, and sustained immunological control [96,97].

In summary, RNA-based vaccines provide an innovative route to treating cancer despite the obvious challenges of optimising vaccine design and refining each combination. New combinations will need to raise response and progression-free survival rates without adding serious toxicity. Based on the low toxicity of mRNA cancer vaccines, they are especially suitable for combination therapies. Figure 3 summarises the four principal combination strategies discussed above together with the representative clinical evidence.

## 6. mRNA Vaccines in Gastrointestinal Cancers: Clinical Trials and Ongoing Studies

The potential scope of mRNA vaccines in GI cancers is very broad [8]. Any interpretation of the trial data, however, must consider several complicating factors. The technologies, mechanisms of action, and delivery platforms differ from one product to another. The “GI field” itself covers tumours that are biologically very dissimilar. Each of these tumours contain multiple molecular subtypes, and the disease setting in which a vaccine is tested (neoadjuvant, adjuvant, metastatic) varies considerably. All these factors affect how far the results of any single trial can be generalised.

For esophageal and gastric cancers, no phase II or III trials have yet been reported. The highest level of evidence comes from phase I studies and a handful of case reports, in which mRNA vaccines are usually combined with immunotherapy [85,98]. A phase I study enrolled patients with advanced esophageal, gastric, PDAC and CRC, while a later report described personalised mRNA vaccination combined with PD-1 inhibition in a patient with advanced esophageal squamous-cell carcinoma [98]. Several exploratory studies of personalised neoantigen vaccines remain registered, including studies of PGV002 in advanced gastric, esophageal, and liver cancers and a separate gastric-cancer study; their registry records do not provide mature efficacy data. Several trials are nonetheless underway (Table 4).

In PDAC, recent progress in mRNA therapeutics has generated considerable interest, and the disease currently has the most active pipeline of gastrointestinal-specific mRNA vaccine studies. In a phase I study of resected PDAC, personalised autogene cevumeran induced de novo neoantigen-specific T-cell responses in eight of sixteen vaccinated patients. Responders had longer recurrence-free survival (RFS) than nonresponders, although this comparison was exploratory and derived from a small, single-arm study without a control group [18]. Moreover, a phase II study involved testing of autogene cevumeran after resection while early-phase studies evaluated iNeo-Vac-R01 in advanced digestive-system tumours, XP-004 in patients unable to tolerate chemotherapy and KRAS-directed vaccines such as ABO2102. Early conference data from iNeo-Vac-R01 combined with first-line chemotherapy reported vaccine-specific T-cell responses in nine of nine evaluable patients, but the study was single-arm and used historical comparisons. Clinical development is still in its early stage, but emerging phase I/II studies have shown encouraging immunogenicity and translational promise [8]. Randomised phase III trials are lacking, and most ongoing studies remain exploratory or early-phase (Table 4).

In colorectal cancer, clinical development has centred on personalised neoantigen vaccines and on KRAS-directed approaches, mainly in metastatic disease. The most advanced study is the randomised phase II GRANITE trial [99]. In this study an immunotherapy regimen combining a chimpanzee adenoviral vector with self-amplifying mRNA encoding patient-specific neoantigens was used as a first-line treatment of MSS colorectal cancer, either alone or added to standard care. Preliminary interim results showed improved progression-free survival compared with standard therapy alone, most clearly in patients with a low baseline disease burden as measured by ctDNA [99]. A higher proportion of patients on the GRANITE arm remained progression-free with undetectable or very low ctDNA during follow-up. This observation suggests better molecular disease control. In addition, neoantigen-specific T-cell responses were seen in all evaluable vaccinated patients, confirming the immunogenicity of the approach [99]. The ongoing trials are listed in Table 4.

## 7. Discussion and Conclusions

The strongest efficacy signal to date comes from melanoma, where the addition of a personalised mRNA vaccine to pembrolizumab in KEYNOTE-942 improved recurrence-free and distant-metastasis-free survival. A favourable, if not yet significant, trend in overall survival at five years has been reported [93,94]. Whether the same premise holds in tumours with a far less favourable immune context is the question that the ongoing GI trials are designed to answer.

Two GI-specific proofs-of-concepts are worth restating because they frame realistic expectations. In resected PDAC, autogene cevumeran induced persistent neoantigen-specific T-cell responses. In addition, recurrence was delayed, and the clonotypes involved were durable rather than transient [18]. This matters in a disease that is almost proverbially resistant to immunotherapy. It suggests that the barrier in PDAC is not an absolute inability to raise an antitumour response. Nevertheless, it is difficult to raise one large and durable enough response to overcome an exceptionally hostile stroma. In CRC, the GRANITE programme has shown that a self-amplifying mRNA and adenoviral regimen can produce measurable molecular responses leading to improved survival in MSS disease. In this setting, checkpoint inhibitors have consistently failed [34,99]. Neither result is yet a phase III endpoint, and it would be premature to treat them as such, but together they justify the current momentum.

Combination, rather than monotherapy, is almost certainly the way these vaccines will be used. The biological argument is based on the following facts. A vaccine expands antigen-specific effector T-cells, but those cells tend to acquire inhibitory markers. To overcome the suppressive microenvironment, combination of the vaccine with checkpoint blockade, and possibly chemotherapy or radiotherapy, is needed to keep immune cells active. Furthermore, the immunogenic cell death supply additional antigens for further T-cell stimulation [18,95,96,97]. Most of the trials listed in Table 4 reflect this thinking, pairing a vaccine with a PD-1 or PD-L1 inhibitor and, in several cases, with cytotoxic chemotherapy. A further important point is the primacy of the disease setting. The adjuvant and minimal-residual-disease settings, where tumour burden is low and the immune system is not yet overwhelmed, appear to be where vaccines perform best. This pattern seems to be a general feature because it was also observed in melanoma patients [93,94] and in the ctDNA-defined subgroups of colon cancer within the GRANITE study [99]. The design of BNT122-01, which randomises ctDNA-positive resected colorectal cancer patients to vaccine or watchful waiting [99], is a direct expression of this logic.

Several limitations exist since almost all the GI data are early-phase, single-arm, or interim, and the endpoints reported—immunogenicity, ctDNA kinetics, and safety—are informative but not definitive. Cross-tumour extrapolation is hazardous, and the heterogeneity of the platforms (conventional versus self-amplifying mRNA, LNP *versus* lipoplex, personalised *versus* shared antigen) means that a positive or negative result with one construct cannot be assumed to generalise to another. The immunosuppressive microenvironment of GI tumours remains the fundamental biological hurdle. It is unlikely that a vaccine alone will improve GI patients` outcome. Nevertheless, combination therapies with mRNA vaccine as one component appear to become a treatment option in the future. Therefore, biomarkers that identify patients most likely to benefit are a prerequisite. Potential biomarkers like TMB, neoantigen load, baseline immune infiltration, or treatment ctDNA dynamics are still being validated and are not yet ready to guide selection in practice.

Looking ahead, progress will probably depend on three parallel efforts. Manufacturing must become faster and cheaper, so that personalised vaccines can be produced within a clinically relevant time. Antigen selection must improve, through better prediction algorithms and, where feasible, a shift towards validated shared targets that can be manufactured in advance. In addition, rational combinations must be tested with attention to the disease setting, prioritising adjuvant and MRD contexts where the immune system has the best chance of success. The maturing phase III data in melanoma and the readouts of the GI trials now underway should, over the next few years, show whether the biological promise seen in early studies can be converted into durable clinical benefit.

In conclusion, mRNA vaccines have introduced a genuinely new paradigm into the treatment of gastrointestinal cancers. Their specificity, adaptability and capacity to raise tumour-specific immunity address the central weakness of current immunotherapy in immunologically cold tumours. The early clinical signals in pancreatic and colorectal cancer are encouraging and biologically coherent. The strong result in melanoma provides a proof-of-principle for the combination strategy that dominates ongoing GI treatments. Considerable work remains—in manufacturing, antigen selection, combination design, and biomarker validation—before these agents can be integrated into standard care. It is highly likely that artificial intelligence (AI) will also speed up the field of mRNA vaccine development. AI can help to identify potential antigens faster and based on biomarker pattern propose the best personalised combination design for patients. The necessary phase III evidence is not yet in hand. Even so, the trajectory of the field, together with the rapid maturation of mRNA technology, suggests that mRNA vaccines are likely to occupy an expanding role in the immunotherapy of gastrointestinal malignancies in the years to come [100,101].

## Figures and Tables

**Table 1:** Comparison of therapeutic cancer-vaccine platforms. Legend: CRC: colorectal cancer; GI: gastrointestinal; HLA: human leucocyte antigen; PDAC: pancreatic ductal adenocarcinoma.

Vaccine Platform	Main Advantages	Main Limitations	Potential Relevance to GI Neoplasia	Representative References
**mRNA vaccines**	Rapid and flexible manufacturing process; can encode multiple tumour antigens or patient-specific neoantigens; transient, non-integrating expression; stimulates both antigen-specific and innate immune responses.	mRNA instability; delivery and biodistribution challenges; excessive innate sensing may reduce translation; manufacturing and quality-control requirements.	Personalised neoantigen vaccination, particularly in PDAC; combination with chemotherapy or immune checkpoint inhibitors; testing in organoid and 3D immune co-culture models.	[16,17,18]
**Peptide or protein** **vaccines**	Chemically defined, relatively stable, and straightforward to manufacture; generally favourable safety profile.	HLA restriction; limited cellular uptake and cross-presentation; often require strong adjuvants and repeated dosing; usually target a limited number of antigens.	Shared or personalised neoantigen vaccines for CRC, PDAC, and other GI tumours.	[19,20,21]
**DNA** **vaccines**	More stable than mRNA; relatively inexpensive and scalable; can support prolonged antigen expression.	Requires entry into the nucleus; delivery can be inefficient; electroporation or specialised vectors may be needed; regulatory concerns related to DNA persistence and genomic integration.	Mainly preclinical or early clinical applications for tumour-associated and neoantigen targets.	[20,21,22]
**Dendritic-cell vaccines**	Directly target professional antigen-presenting cells; can use peptides, tumour lysates, or mRNA; allow ex vivo quality control.	Patient-specific manufacturing is labour-intensive, expensive, and difficult to standardise; dendritic cells may become dysfunctional in an immunosuppressive tumour environment.	Personalised vaccination and combination strategies in PDAC, CRC, and other GI cancers.	[19,20]
**Viral-vector vaccines**	Efficient antigen delivery and strong innate and adaptive immune activation; high intracellular antigen expression.	Pre-existing or therapy-induced anti-vector immunity; limited repeat dosing; cargo constraints; vector-specific safety concerns.	Useful for tumour-associated antigens, neoantigens, and oncolytic strategies, although clinical translation remains context-dependent.	[21,22]
**Whole-cell or tumour-** **lysate** **vaccines**	Broad antigen repertoire; do not require complete prior identification of individual neoantigens; may address tumour heterogeneity.	Poorly defined antigen composition; variable potency and manufacturing; difficult to identify the active components; inconsistent reproducibility.	Exploratory personalised approaches for heterogeneous GI tumours and postoperative minimal-residual-disease settings.	[20,22]

**Table 2:** Structural elements of mRNA vaccines.

Element	Function
5′ cap	Protects the mRNA from degradation and enables the initiation of translation
5′ UTR (untranslated region)	Controls the start of translation; vaccines typically use short UTRs containing a Kozak sequence
Coding region	Contains the sequence of the target antigen (e.g., the SARS-CoV-2 spike protein)
3′ UTR	Regulates transcript stability; the α-globin UTR is frequently used to improve it
Poly(A) tail	Improves stability and translation; longer tails are generally associated with higher expression

**Table 3:** Mechanistic components of mRNA vaccine activity in GI cancers.

Mechanistic Stage	Principal Events	Antitumour Consequence	Representative Evidence
Delivery and translation	LNP or liposomal uptake, endosomal escape, cytoplasmic translation	Production of tumour antigens without genomic integration	[19,23,44]
Innate sensing	TLR, RIG-I, and MDA5 activation; type I interferon and NF-κB signalling	Dendritic-cell maturation and inflammatory conditioning	[60,61,80]
Antigen presentation	MHC class I cross-presentation and MHC class II presentation	CD8^+^ cytotoxic and CD4^+^ helper T-cell priming	[19,82]
TME remodelling	CXCL9, CXCL10, and CXCL11 induction; increased dendritic-cell and CD8^+^ T-cell infiltration	Conversion of immune-excluded tumours into T-cell-inflamed tumours	[83,84]
Checkpoint cooperation	Expansion of tumour-reactive T-cells alongside PD-1 and PD-L1 induction	Improved response to PD-1 or CTLA-4 blockade	[80,83,85]
Memory and durability	Polyfunctional effector cells, progenitor-exhausted T-cells, clonal persistence, and booster responses	Prolonged immune surveillance and possible recurrence prevention	[18,33,85]
Limitations	Neoantigen heterogeneity, poor tumour access, excessive innate sensing, and immunosuppressive TME	Immunogenicity without sufficient tumour control	[18,69]

Legend: LNP: lipid nanoparticle; MHC: major histocompatibility complex; TLR: toll-like receptor.

**Table 4:** Main ongoing clinical trials of mRNA vaccines in gastrointestinal tumours. Legend: NR: not reported; PD-1: programmed death protein 1; PD-L1: programmed death-ligand 1; NSCLC: non-small cell lung cancer; TTP: time to progression; DCR: disease control rate; ORR: objective response rate; OS: overall survival; PDAC: pancreatic ductal adenocarcinoma; RFS: recurrence-free survival; PK: pharmacokinetics; LNP: lipid nanoparticle; MRD: minimal residual disease; ctDNA: circulating tumour DNA; CRC: colorectal cancer; pMMR: proficient mismatch repair; GI: gastrointestinal; PFS: progression-free survival; ICI: immune checkpoint inhibitor.

Tumour Type/Setting	NCT ID/Trial Name	Phase/Status/Target	mRNA Strategy	Arms	Key Objectives/Endpoints
Advanced gastric, esophageal and liver cancer	NCT05192460	Phase NR; recruiting; ~30 pts	Personalised neoantigen mRNA vaccine PGV002	PGV002 alone; expansion with PD-1/PD-L1 inhibitor	Exploratory study. Safety, dose selection, ORR, PFS, cytokines and lymphocyte responses.
Advanced esophageal cancer and NSCLC	NCT03908671	Phase I-type; recruiting; ~24 pts	Personalised neoantigen mRNA tumour vaccine	mRNA vaccine after standard-treatment failure/no standard option	Single-arm study. Safety/tolerability; TTP, DCR, ORR, and OS.
Advanced unresectable/metastatic PDAC	NCT06888648	Phase I/II; not yet recruiting	Personalised tumour neoantigen mRNA therapy, iNeo-Vac-R01	iNeo-Vac-R01 with FOLFIRINOX components and sintilimab	Early feasibility and safety.
Postoperative PDAC, chemotherapy-intolerant	NCT06496373	Early Phase I; recruiting	Personalised mRNA neoantigen vaccine XP-004	XP-004 + PD-1 inhibitor	Safety/tolerability; neoantigen-specific CD4/CD8 responses, RFS, and OS.
KRAS-mutant advanced PDAC and other solid tumours	NCT06577532	Early Phase I; recruiting; ~56 pts	Shared KRAS-mutant neoantigen mRNA vaccine ABO2102	ABO2102 alone or + toripalimab	Dose escalation and expansion study. Safety, immunogenicity, pharmacodynamics, and preliminary efficacy.
Resected PDAC	NCT06353646	Phase NR; recruiting; ~24 pts	Neoantigen cancer vaccine XH001 (mRNA nature reported in secondary sources)	XH001 sequentially with ICI and chemotherapy	Safety and efficacy.
Resected PDAC	NCT04161755	Phase I; completed or follow-up ongoing	Autogene cevumeran (BNT122), personalised uridine mRNA–lipoplex,	BNT122 with atezolizumab and mFOLFIRINOX	Single-arm study of safety, feasibility, immunogenicity, and recurrence. 8/16 vaccinated patients developed strong neoantigen-specific T-cell responses [18].
Resected PDAC	NCT05968326, IMCODE003	Phase II; recruiting or actively enrolling	Autogene cevumeran (BNT122) plus atezolizumab and mFOLFIRINOX versus mFOLFIRINOX alone	BNT122 plus atezolizumab and mFOLFIRINOX versus mFOLFIRINOX alone	Multicentre randomised study assessing disease-free survival, overall survival, safety, and immunogenicity. This is the principal confirmatory trial arising from the phase I PDAC findings.
Resected PDAC	NCT06156267	Early Phase I; active, not recruiting; ~30 pts	Personalised neoantigen mRNA tumour vaccine	mRNA vaccine + adebrelimab	Safety/tolerability.
Surgically resected PDAC	NCT06913218	Phase I; active, not recruiting; ~60 pts	mRNA vaccine AK154	AK154 alone or + AK104/AK112, followed by mFOLFIRINOX	Safety, tolerability, efficacy, immunogenicity, PK/pharmacodynamics.
ctDNA-positive resected stage II high-risk/stage III CRC	NCT04486378/BNT122-01	Phase II recruitment complete, follow-up ongoing	Individualised neoantigen mRNA vaccine RO7198457/BNT122	RO7198457 vs. watchful waiting after standard adjuvant chemotherapy	Vaccine versus watchful waiting after standard adjuvant chemotherapy. Efficacy in ctDNA-positive MRD setting.
First-line metastatic MSS CRC	NCT05141721, GRANITE	Phase II/III; phase II analysis reported; later-stage study ongoing	GRT-C901 + GRT-R902	GRT-C901 + GRT-R902 + atezolizumab + ipilimumab + bevacizumab and fluoropyrimidine vs. bevacizumab and fluoropyrimidine	Randomised comparison with standard therapy alone. Interim PFS favoured GRANITE, but the confidence interval crossed unity; the strongest signal was in patients with lower baseline ctDNA burden.
KRAS-mutated advanced malignancies, incl. CRC/PDAC	NCT07004244	Phase I; recruiting; ~20 pts	KRAS-mutated mRNA vaccine	KRAS mRNA vaccine + toripalimab ± chemotherapy	Single-arm study assessing safety, disease control, PFS, TTP, and OS.
Advanced pMMR CRC with liver metastases	NCT07610707	Phase I; not yet recruiting; ~10 pts	YMN-A02, LNP-mRNA encoding CTLA-4 antibody and TGF-β trap bifunctional protein	YMN-A02 dose escalation	Safety, tolerability, immunogenicity, preliminary activity.
PD-1/PD-L1-resistant advanced solid tumours, incl. gastric cancer	NCT05949775	Early phase; not yet recruiting	Neoantigen mRNA tumour vaccine	mRNA vaccine ± PD-1/PD-L1 inhibitor	Safety and preliminary efficacy.
Advanced MSS CRC and gastroesophageal adenocarcinoma	NCT03639714	Phase I; reported	GRT-C901 chimpanzee adenoviral prime plus GRT-R902 self-amplifying mRNA boost	GRT-C901 + GRT-R902 with nivolumab with or without ipilimumab	7 MSS-CRC and 6 gastroesophageal patients were treated. Broad T-cell responses were observed.
Advanced GI neoplasms	NCT06019702	Phase I; recruiting	Personalised neoantigen mRNA vaccine iNeo-Vac-R01	iNeo-Vac-R01 monotherapy	Dose-escalation and expansion study assessing safety, feasibility, ORR, PFS, OS, and immunogenicity.
Advanced GI neoplasms, first-line setting	NCT06026800	Phase I; recruiting	Personalised neoantigen mRNA vaccine iNeo-Vac-R01	iNeo-Vac-R01 + standard first-line treatment	Single-arm feasibility, safety, efficacy, and immunogenicity study. Conference data reported T-cell responses in 9 of 9 evaluable patients.
Resected GI neoplasms	NCT06026774	Phase I; recruiting	Personalised neoantigen mRNA vaccine iNeo-Vac-R01	iNeo-Vac-R01 + standard adjuvant therapy	Single-arm study assessing safety, feasibility, recurrence, survival, and immune responses across multiple gastrointestinal primary sites.
GI tumours	NCT07067385	Phase I; recruiting	Neoantigen personalised mRNA cancer vaccine deepGeneAI-001	deepGeneAI-001 + sintilimab	Efficacy and safety.

## Data Availability

No new data were created or analysed in this study. Data sharing is not applicable to this article.

## References

[1] Bray F., Laversanne M., Sung H., Ferlay J., Siegel R.L., Soerjomataram I., Jemal A. (2024). Global cancer statistics 2022: GLOBOCAN estimates of incidence and mortality worldwide for 36 cancers in 185 countries. CA Cancer J. Clin..

[2] Chen D.S., Mellman I. (2013). Oncology meets immunology: The cancer-immunity cycle. Immunity.

[3] Galon J., Bruni D. (2020). Tumour immunology and tumour evolution: Intertwined histories. Immunity.

[4] Le D.T., Durham J.N., Smith K.N., Wang H., Bartlett B.R., Aulakh L.K., Lu S., Kemberling H., Wilt C., Luber B.S. (2017). Mismatch repair deficiency predicts response of solid tumours to PD-1 blockade. Science.

[5] Bear A.S., Vonderheide R.H., O’Hara M.H. (2020). Challenges and opportunities for pancreatic cancer immunotherapy. Nat. Rev. Clin. Oncol..

[6] Melief C.J.M., van Hall T., Arens R., Ossendorp F., van der Burg S.H. (2015). Therapeutic cancer vaccines. J. Clin. Investig..

[7] Wang Y., Huang P., Li C., Tu S., Yang H. (2025). Nanovaccines in gastrointestinal cancers. Front. Immunol..

[8] Saha S., Chakraborty S., Nayak A. (2026). mRNA vaccines in gastrointestinal cancers: Mechanistic basis, translational challenges, and emerging therapeutic strategies. Crit. Rev. Oncol. Hematol..

[9] Li J., Liu Y., Dai J., Yang L., Xiong F., Xia J., Jin J., Wu Y., Peng X. (2025). mRNA vaccines: Current applications and future directions. MedComm.

[10] Sahin U., Karikó K., Türeci Ö. (2014). mRNA-based therapeutics—Developing a new class of drugs. Nat. Rev. Drug Discov..

[11] Schlake T., Thess A., Fotin-Mleczek M., Kallen K.J. (2019). Developing mRNA-vaccine technologies. Mol. Ther..

[12] Sahin U., Derhovanessian E., Miller M., Kloke B.-P., Simon P., Löwer M., Bukur V., Tadmor A.D., Luxemburger U., Schrörs B. (2017). Personalized RNA mutanome vaccines mobilize poly-specific therapeutic immunity against cancer. Nature.

[13] Kranz L.M., Diken M., Haas H., Kreiter S., Loquai C., Reuter K.C., Meng M., Fritz D., Vascotto F., Hefesha H. (2016). Systemic RNA delivery to dendritic cells exploits antiviral defence for cancer immunotherapy. Nature.

[14] Weide B., Carralot J.-P., Reese A., Scheel B., Eigentler T.K., Hoerr I., Rammensee H.-G., Garbe C., Pascolo S. (2008). Results of the First Phase I/II Clinical Vaccination Trial with Direct Injection of mRNA. J. Immunother..

[15] Taumar D., Singh A.P., Sharma H., Gujjar A., Rajpoot I., Chaudhary V. (2026). Nano-vaccines in pandemic preparedness: Pioneering rapid development and deployment for global immunity. Curr. Gene Ther..

[16] Miao L., Zhang Y., Huang L. (2021). mRNA vaccine for cancer immunotherapy. Mol. Cancer.

[17] Jacob E.M., Huang J., Chen M. (2024). Lipid nanoparticle-based mRNA vaccines: A new frontier in precision oncology. Precis. Clin. Med..

[18] Rojas L.A., Sethna Z., Soares K.C., Olcese C., Pang N., Patterson E., Lihm J., Ceglia N., Guasp P., Chu A. (2023). Personalized RNA neoantigen vaccines stimulate T cells in pancreatic cancer. Nature.

[19] Esprit A., de Mey W., Bahadur Shahi R., Thielemans K., Franceschini L., Breckpot K. (2020). Neo-Antigen mRNA Vaccines. Vaccines.

[20] Guo X., Zhang Y., Zheng L., Zheng C., Song J., Zhang Q., Kang B., Liu Z., Jin L., Xing R. (2018). Global characterization of T cells in non-small-cell lung cancer by single-cell sequencing. Nat. Med..

[21] Shemesh C.S., Hsu J.C., Hosseini I., Shen B.Q., Rotte A., Twomey P., Girish S., Wu B. (2021). Personalized Cancer Vaccines: Clinical Landscape, Challenges, and Opportunities. Mol. Ther..

[22] Fritah H., Rovelli R., Chiang C.L.-L., Kandalaft L.E. (2022). The current clinical landscape of personalized cancer vaccines. Cancer Treat. Rev..

[23] Hou X., Zaks T., Langer R., Dong Y. (2021). Lipid nanoparticles for mRNA delivery. Nat. Rev. Mater..

[24] Yaremenko A.V., Khan M.M., Zhen X., Tang Y., Tao W. (2025). Clinical advances of mRNA vaccines for cancer immunotherapy. Med.

[25] Zaidi N., Jaffee E.M., Yarchoan M. (2025). Recent advances in therapeutic cancer vaccines. Nat. Rev. Cancer.

[26] Iavarone C., O’Hagan D.T., Yu D., Delahaye N.F., Ulmer J.B. (2017). Mechanism of action of mRNA-based vaccines. Expert Rev. Vaccines.

[27] Wolff J.A., Malone R.W., Williams P., Chong W., Acsadi G., Jani A., Felgner P.L. (1990). Direct gene transfer into mouse muscle in vivo. Science.

[28] Martinon F., Krishnan S., Lenzen G., Magné R., Gomard E., Guillet J.G., Lévy J.P., Meulien P. (1993). Induction of virus-specific cytotoxic T lymphocytes in vivo by liposome-entrapped mRNA. Eur. J. Immunol..

[29] Hoerr I., Obst R., Rammensee H.G., Jung G. (2000). In vivo application of RNA leads to induction of specific cytotoxic T lymphocytes and antibodies. Eur. J. Immunol..

[30] O’Brien J., Hayder H., Zayed Y., Peng C. (2018). Overview of microRNA biogenesis, mechanisms of actions, and circulation. Front. Endocrinol..

[31] Chaudhary N., Weissman D., Whitehead K.A. (2021). mRNA vaccines for infectious diseases: Principles, delivery and clinical translation. Nat. Rev. Drug Discov..

[32] Bettini E., Locci M. (2021). SARS-CoV-2 mRNA vaccines: Immunological mechanism and beyond. Vaccines.

[33] Sethna Z., Guasp P., Reiche C., Milighetti M., Ceglia N., Patterson E., Lihm J., Payne G., Lyudovyk O., Rojas L.A. (2025). RNA neoantigen vaccines prime long-lived CD8+ T cells in pancreatic cancer. Nature.

[34] Hecht J.R., Shergill A., Goldstein M.G., Fang B., Cho M.T., Lenz H.-J., Berim L.D., Oberstein P.E., A Safyan R., Sawhney V. (2022). Phase 2/3, randomized, open-label study of an individualized neoantigen vaccine (self-amplifying mRNA and adenoviral vectors) plus immune checkpoint blockade as maintenance for patients with newly diagnosed metastatic colorectal cancer (GRANITE). J. Clin. Oncol..

[35] Sittplangkoon C., Alameh M.G., Weissman D., Lin P.J.C., Tam Y.K., Prompetchara E., Palaga T. (2022). mRNA vaccine with unmodified uridine induces robust type I interferon-dependent anti-tumour immunity in a melanoma model. Front. Immunol..

[36] Mulroney T.E., Pöyry T., Yam-Puc J.C., Rust M., Harvey R.F., Kalmar L., Horner E., Booth L., Ferreira A.P., Stoneley M. (2024). N1-methylpseudouridylation of mRNA causes +1 ribosomal frameshifting. Nature.

[37] Zhang A., Ji Q., Sheng X., Wu H. (2023). mRNA vaccine in gastrointestinal tumours: Immunomodulatory effects and immunotherapy. Biomed. Pharmacother..

[38] Gergen J., Petsch B., Yu D., Petsch B. (2020). mRNA-based vaccines and mode of action. mRNA Vaccines.

[39] Stitz L., Vogel A., Schnee M., Voss D., Rauch S., Mutzke T., Ketterer T., Kramps T., Petsch B. (2017). A thermostable messenger RNA based vaccine against rabies. PLoS Negl. Trop. Dis..

[40] Schäfer M.E.A., Keller F., Schumacher J., Haas H., Vascotto F., Sahin U., Hafner M., Rudolf R. (2022). 3D Melanoma Cocultures as Improved Models for Nanoparticle-Mediated Delivery of RNA to Tumors. Cells.

[41] Subtil B., Iyer K.K., Poel D., Bakkerus L., Gorris M.A.J., Escalona J.C., Dries K., Cambi A., Verheul H.M.W., de Vries I.J.M. (2023). Dendritic cell phenotype and function in a 3D co-culture model of patient-derived metastatic colorectal cancer organoids. Front. Immunol..

[42] Le Bras A. (2024). New insights into mRNA vaccine-triggered immunity. Lab Anim..

[43] Cagigi A., Loré K. (2021). Immune responses induced by mRNA vaccination in mice, monkeys and humans. Vaccines.

[44] Xu S., Yang K., Li R., Zhang L. (2020). mRNA vaccine era—Mechanisms, drug platform and clinical prospection. Int. J. Mol. Sci..

[45] Overmars I., Au-Yeung G., Nolan T.M., Steer A.C. (2022). mRNA vaccines: A transformative technology with applications beyond COVID-19. Med. J. Aust..

[46] Bar-On L., Cohen H., Elia U., Cherry-Mimran L., Cohen O., Erez N. (2025). The mRNA component of LNP-mRNA vaccines triggers IFNAR-dependent immune activation which attenuates the adaptive immune response. Front. Immunol..

[47] Medjmedj A., Genon H., Hezili D., Ngalle Loth A., Clemençon R., Guimpied C., Mollet L., Bigot A., Wien F., Hamacek J. (2025). Evaluation of synthetic mRNA with selected UTR sequences and alternative poly(A) tail, in vitro and in vivo. Mol. Ther. Nucleic Acids.

[48] Lee K.H., Lee J., Lee S.-W. (2026). Circular RNA as a new vaccine platform: Considerations, challenges, and perspectives. Vaccines.

[49] Li W., Li Y., Li J., Meng J., Jiang Z., Yang C., Wen Y., Liu S., Cheng X., Mi S. (2024). All-Trans-Retinoic Acid-Adjuvanted mRNA Vaccine Induces Mucosal Anti-Tumor Immune Responses for Treating Colorectal Cancer. Adv. Sci..

[50] Gote V., Bolla P.K., Kommineni N., Butreddy A., Nukala P.K., Palakurthi S.S., Khan W. (2023). A comprehensive review of mRNA vaccines. Int. J. Mol. Sci..

[51] Zhang Z., Du J., Zhang D., Han R., Wu X., Liang Y. (2025). Research progress of mRNA vaccines for infectious diseases. Eur. J. Med. Res..

[52] Frei J., Eisel D., Jarzebska N.T., Mellett M., Yener I.G., Abdou M.T., Gouttefangeas C., Flatz L., Diken M., Kündig T.M. (2026). Chemically synthesized, non-capped and non-polyadenylated peptide-coding RNA efficiently induces antigen-specific CD8^+^ T cells. Nat. Biomed. Eng..

[53] Saxena M., van der Burg S.H., Melief C.J.M., Bhardwaj N. (2021). Therapeutic cancer vaccines. Nat. Rev. Cancer.

[54] Ott P.A., Hu-Lieskovan S., Chmielowski B., Govindan R., Naing A., Bhardwaj N., Margolin K., Awad M.M., Hellmann M.D., Lin J.J. (2020). A phase Ib trial of personalized neoantigen therapy plus anti-PD-1. Cell.

[55] Verbeke R., Lentacker I., De Smedt S.C., Dewitte H. (2021). The dawn of mRNA vaccines: The COVID-19 case. J. Control. Release.

[56] Pardi N., Hogan M.J., Porter F.W., Weissman D. (2018). mRNA vaccines—A new era in vaccinology. Nat. Rev. Drug Discov..

[57] Karikó K., Buckstein M., Ni H., Weissman D. (2005). Suppression of RNA recognition by Toll-like receptors: The impact of nucleoside modification and the evolutionary origin of RNA. Immunity.

[58] Karikó K., Muramatsu H., Welsh F.A., Ludwig J., Kato H., Akira S., Weissman D. (2008). Incorporation of pseudouridine into mRNA yields superior nonimmunogenic vector with increased translational capacity and biological stability. Mol. Ther..

[59] Thess A., Grund S., Mui B.L., Hope M.J., Baumhof P., Fotin-Mleczek M., Schlake T. (2015). Sequence-engineered mRNA without chemical nucleoside modifications enables an effective protein therapy in large animals. Mol. Ther..

[60] Kawai T., Akira S. (2010). The role of pattern-recognition receptors in innate immunity. Nat. Immunol..

[61] Iwasaki A., Medzhitov R. (2010). Regulation of adaptive immunity by the innate immune system. Science.

[62] Pardi N., Hogan M.J., Weissman D. (2020). Recent advances in mRNA vaccine technology. Curr. Opin. Immunol..

[63] Ott P.A., Hu Z., Keskin D.B., Shukla S.A., Sun J., Bozym D.J., Zhang W., Luoma A., Giobbie-Hurder A., Peter L. (2017). An immunogenic personal neoantigen vaccine for patients with melanoma. Nature.

[64] Binnewies M., Roberts E.W., Kersten K., Chan V., Fearon D.F., Merad M., Coussens L.M., Gabrilovich D.I., Ostrand-Rosenberg S., Hedrick C.C. (2018). Understanding the tumour immune microenvironment (TIME) for effective therapy. Nat. Med..

[65] Ramirez F., Zambrano A., Hennis R., Holland N., Lakshmanaswamy R., Chacon J. (2023). Sending a message: Use of mRNA vaccines to target the tumor immune microenvironment. Vaccines.

[66] Melero I., Castanon E., Alvarez M., Champiat S., Marabelle A. (2021). Intratumoural administration and tumour tissue targeting of cancer immunotherapies. Nat. Rev. Clin. Oncol..

[67] Zamarin D., Holmgaard R.B., Ricca J., Plitt T., Palese P., Sharma P., Merghoub T., Wolchok J.D., Allison J.P. (2017). Intratumoural modulation of the inducible co-stimulator ICOS by recombinant oncolytic virus promotes systemic anti-tumour immunity. Sci. Transl. Med..

[68] Hilf N., Kuttruff-Coqui S., Frenzel K., Bukur V., Stevanović S., Gouttefangeas C., Platten M., Tabatabai G., Dutoit V., Van Der Burg S.H. (2019). Actively personalized vaccination trial for newly diagnosed glioblastoma. Nature.

[69] Cafri G., Gartner J.J., Zaks T., Hopson K., Levin N., Paria B.C., Parkhurst M.R., Yossef R., Lowery F.J., Jafferji M.S. (2020). mRNA vaccine-induced neoantigen-specific T cell immunity in patients with gastrointestinal cancer. J. Clin. Investig..

[70] Rizvi N.A., Hellmann M.D., Snyder A., Kvistborg P., Makarov V., Havel J.J., Lee W., Yuan J., Wong P., Ho T.S. (2015). Mutational landscape determines sensitivity to PD-1 blockade in non-small cell lung cancer. Science.

[71] Pant S., Wainberg Z.A., Weekes C.D., Furqan M., Kasi P.M., Devoe C.E., Leal A.D., Chung V., Basturk O., VanWyk H. (2024). Lymph-node-targeted, mKRAS-specific amphiphile vaccine in pancreatic and colorectal cancer: The phase 1 AMPLIFY-201 trial. Nat. Med..

[72] Canon J., Rex K., Saiki A.Y., Mohr C., Cooke K., Bagal D., Gaida K., Holt T., Knutson C.G., Koppada N. (2019). The clinical KRAS(G12C) inhibitor AMG 510 drives anti-tumour immunity. Nature.

[73] Lopez J., Powles T., Braiteh F., Siu L.L., LoRusso P., Friedman C.F., Balmanoukian A.S., Gordon M., Yachnin J., Rottey S. (2025). Autogene cevumeran with or without atezolizumab in advanced solid tumors: A phase 1 trial. Nat. Med..

[74] Goodman A.M., Kato S., Bazhenova L., Patel S.P., Frampton G.M., Miller V., Stephens P.J., Daniels G.A., Kurzrock R. (2017). Tumour mutational burden as an independent predictor of response to immunotherapy in diverse cancers. Mol. Cancer Ther..

[75] McGranahan N., Furness A.J.S., Rosenthal R., Ramskov S., Lyngaa R., Saini S.K., Jamal-Hanjani M., Wilson G.A., Birkbak N.J., Hiley C.T. (2016). Clonal neoantigens elicit T cell immunoreactivity and sensitivity to immune checkpoint blockade. Science.

[76] Yost K.E., Satpathy A.T., Wells D.K., Qi Y., Wang C., Kageyama R., McNamara K.L., Granja J.M., Sarin K.Y., Brown R.A. (2019). Clonal replacement of tumour-specific T cells following PD-1 blockade. Nat. Med..

[77] Wu S.Z., Al-Eryani G., Roden D.L., Junankar S., Harvey K., Andersson A., Thennavan A., Wang C., Torpy J.R., Bartonicek N. (2021). A single-cell and spatially resolved atlas of human breast cancers. Nat. Genet..

[78] Sade-Feldman M., Yizhak K., Bjorgaard S.L., Ray J.P., de Boer C.G., Jenkins R.W., Lieb D.J., Chen J.H., Frederick D.T., Barzily-Rokni M. (2018). Defining T cell states associated with response to checkpoint immunotherapy in melanoma. Cell.

[79] Helmink B.A., Reddy S.M., Gao J., Zhang S., Basar R., Thakur R., Yizhak K., Sade-Feldman M., Blando J., Han G. (2020). B cells and tertiary lymphoid structures promote immunotherapy response. Nature.

[80] Heidegger S., Kreppel D., Bscheider M., Stritzke F., Nedelko T., Wintges A., Bek S., Fischer J.C., Graalmann T., Kalinke U. (2019). RIG-I activating immunostimulatory RNA boosts the efficacy of anticancer vaccines and synergizes with immune checkpoint blockade. eBioMedicine.

[81] Mendez-Gomez H.R., DeVries A., Castillo P., von Roemeling C., Qdaisat S., Stover B.D., Xie C., Weidert F., Zhao C., Moor R. (2024). RNA aggregates harness the danger response for potent cancer immunotherapy. Cell.

[82] Cho S., Kwak W., Yoon H., Lee J., Lee S., Park H., Jo S., Lee Y., Seo Y., Cho Y. (2025). Rapid-Turnaround Co-Administration of mRNA-Based MHC-I and MHC-II-Restricted Neoantigens Enhances Immune Responses of Antigen-Specific CD8^+^ T Cells and Anti-Cancer Efficacy in Colorectal Cancer. Adv. Sci..

[83] Fournier C., Mercey-Ressejac M., Derangère V., Al Kadi A., Rageot D., Charrat C., Leroy A., Vollaire J., Josserand V., Escudé M. (2025). Nanostructured lipid carriers based mRNA vaccine leads to a T cell–inflamed tumour microenvironment favourable for improving PD-1/PD-L1 blocking therapy and long-term immunity in a cold tumour model. eBioMedicine.

[84] Tanji Y., Shimada S., Kato M., Akiyama Y., Hatano M., Tsukihara S., Igarashi Y., Kodera K., Okazaki K., Yasukawa K. (2026). Cytokine mRNA-based therapy alleviates dendritic cell and T cell paucity to eliminate aggressive pancreatic cancer in preclinical mouse models. eBioMedicine.

[85] Nagaoka K., Nakanishi H., Tanaka H., Anindita J., Kawamura T., Tanaka T., Yamashita T., Kuroda A., Nomura S., Akita H. (2025). Neoantigen mRNA vaccines induce progenitor-exhausted T cells that support anti-PD-1 therapy in gastric cancer with peritoneal metastasis. Gastric Cancer.

[86] Li J., Wang P. (2025). Advances in cancer vaccines for digestive system cancers: A systematic analysis of clinical trials. Cancer Manag. Res..

[87] Abu Harirah H.A., Mohammad S.I., Vasudevan A., Jain V., Uthirapathy S., Ganesan S., Bhanot D., Naidu K.S., Mustafa Y.F., Al-Qaim Z.H. (2025). mRNA vaccines for gastrointestinal cancers: A paradigm shift in treatment. Naunyn-Schmiedeberg’s Arch. Pharmacol..

[88] Keivany M.R., Asrami E.S., Besharatloo M., Latifi H., Barjasteh A.H. (2026). Advances in immunotherapy for colorectal cancer: Overcoming resistance in mismatch repair-proficient tumours. Cancer Cell Int..

[89] Li J.Y., Jiang R.Y., Wang J., Wang X.J. (2025). Advances in mRNA vaccine therapy for breast cancer research. Discov. Oncol..

[90] Laflin A.D., Pan J., You M., Jiang S. (2026). Recent advances in mRNA therapeutic cancer vaccines. Annu. Rev. Biomed. Eng..

[91] Li X., Kaboli P.J., Roozitalab G., Salahi S., Qian H., Zhang X., Chen K., Imani S. (2026). Next-generation neoantigen mRNA vaccines: Immuno-engineering strategies for precision cancer immunotherapy. Cell. Oncol..

[92] Gallio C., Esposito L., Passardi A. (2025). Therapeutic cancer vaccines in colorectal cancer: Platforms, Mechanisms and Combinations. Cancers.

[93] Khattak A., Carlino M.S., Meniawy T., Ansstas G., Taylor M.H., Kim K.B., McKean M., Long G.V., Sullivan R.J., Faries M. (2026). Intismeran autogene plus pembrolizumab versus pembrolizumab alone in high-risk resected melanoma: 5-year update of the randomized phase 2b KEYNOTE-942 study. J. Clin. Oncol..

[94] Weber J.S., Carlino M.S., Khattak A., Meniawy T., Ansstas G., Taylor M.H., Kim K.B., McKean M., Long G.V., Sullivan R.J. (2024). Individualised neoantigen therapy mRNA-4157 (V940) plus pembrolizumab versus pembrolizumab monotherapy in resected melanoma (KEYNOTE-942): A randomised, phase 2b study. Lancet.

[95] Yang B., Liu J., Li Y., Liu X. (2025). mRNA cancer vaccines: From pandemic paradigm to personalized oncology therapeutics. Cancer Innov..

[96] Huang K.C., Chen W.T., Chen J.Y., Lee C.Y., Wu C.H., Lai C.Y., Yang P.C., Liang J.A., Shiau A.C., Chao K.S.C. (2024). Neoantigen-augmented iPSC cancer vaccine combined with radiotherapy promotes antitumour immunity in poorly immunogenic cancers. npj Vaccines.

[97] Lhuillier C., Rudqvist N.P., Yamazaki T., Zhang T., Charpentier M., Galluzzi L., Dephoure N., Clement C.C., Santambrogio L., Zhou X.K. (2021). Radiotherapy-exposed CD8+ and CD4+ neoantigens enhance tumour control. J. Clin. Investig..

[98] Wang B., Peng X., Li J., Wang Y., Chen L., Wu M., Zhang Y., Wang W., Feng D., Tang S. (2024). Personalized mRNA vaccine combined with PD-1 inhibitor therapy in a patient with advanced esophageal squamous cell carcinoma. Am. J. Cancer Res..

[99] Hecht J.R., Spira A.I., Nguyen A.V., Berim L.D., Starodub A., Pelster M., Gallinson D., Kasi A., Reilley M., Pimentel A. (2025). A randomized phase 2 study of an individualized neoantigen-targeting immunotherapy in patients with newly diagnosed metastatic microsatellite stable colorectal cancer (MSS-CRC). J. Clin. Oncol..

[100] Pail O., Lin M.J., Anagnostou V., Brown B.D., Brody J.D. (2025). Cancer vaccines and the future of immunotherapy. Lancet.

[101] Leong K.Y., Tham S.K., Poh C.L. (2025). Revolutionizing immunization: A comprehensive review of mRNA vaccine technology and applications. Virol. J..

